
JOURNAL INFORMATION
==============================
NLM Title Abbreviation: Wirtschaftsdienst
Journal ID: Wirtschaftsdienst
NLM Journal ID: 101086612

Wirtschaftsdienst (Hamburg, Germany : 1949)

ISSN: 0043-6275

ARTICLE INFORMATION
==============================
PMCID: PMC7237618
PMID: 32454542
DOI: 10.1007/s10273-020-2649-8
Article ID: 2649
Article version: 1
Subjects: Zeitgespräch

Institutionelle Aspekte einer neuen Handelsordnung

Poitiers Niclas Frederic niclas.poitiers@bruegel.org

grid.432713.0 Bruegel, Rue de la Charité 33, 1210 Saint-Josse-ten-Noode, Belgien
Electronic publication date: 2020 May 20
Print publication date: 2020
Volume: 100
Issue: 5
First page: 328
Last page: 332
Copyright: © Der/die Autor(en) 2020
License: Open Access Dieser Artikel wird unter der Creative Commons Namensnennung 4.0 International Lizenz veröffentlicht, welche die Nutzung, Vervielfältigung, Bearbeitung, Verbreitung und Wiedergabe in jeglichem Medium und Format erlaubt, sofern Sie den/die ursprünglichen Autor(en) und die Quelle ordnungsgemäß nennen, einen Link zur Creative Commons Lizenz beifügen und angeben, ob Änderungen vorgenommen wurden.
Die in diesem Artikel enthaltenen Bilder und sonstiges Drittmaterial unterliegen ebenfalls der genannten Creative Commons Lizenz, sofern sich aus der Abbildungslegende nichts anderes ergibt. Sofern das betreffende Material nicht unter der genannten Creative Commons Lizenz steht und die betreffende Handlung nicht nach gesetzlichen Vorschriften erlaubt ist, ist für die oben aufgeführten Weiterverwendungen des Materials die Einwilligung des jeweiligen Rechteinhabers einzuholen.
Weitere Details zur Lizenz entnehmen Sie bitte der Lizenzinformation auf http://creativecommons.org/licenses/by/4.0/deed.de.
License URL: https://creativecommons.org/licenses/by/4.0/

issue-copyright-statement: © The Author(s) 2020

==============================

Die internationale Handelsordnung steht vor gewaltigen Herausforderungen. Der wirtschaftliche Aufstieg Chinas und die Abwanderung des Verarbeitenden Gewerbes aus Industrienationen haben mit dazu geführt, dass China in den USA immer mehr als „Systemgegner" gesehen wird. Konservative China-Falken in den USA sprechen bereits von einem „technologischen Kalten Krieg" (Crabtree et al., 2019). Im Januar 2020 hat der Handelskrieg des US-Präsidenten Trump gegen China zunächst durch das „Phase-1-Abkommen" ein Art Waffenstillstand erreicht. Doch dieses Abkommen löst keine der Grundursachen des Konflikts. Selbst vor der SARS-CoV-2-Pandemie war es hoch fragwürdig, ob die Zusagen, die China in diesem Abkommen gemacht hat, erreichbar waren (Bown, 2020). Diese sind jetzt endgültig nicht mehr einzuhalten, und die wichtigsten Beschwerden der USA gegenüber China sind in dem Abkommen noch gar nicht adressiert worden. Ein Kollateralschaden dieses Handelskriegs erleidet die internationale Wirtschaftsordnung, da die USA internationale Regeln zur Verhinderung von Zöllen ignoriert und die Schiedsgerichtsbarkeit der Welthandelsorganisation (WTO) lahmgelegt haben.

Nun verstärkt die SARS-CoV-2-Pandemie die negativen Trends im weltweiten Handel weiter. Die Prognosen des Internationalen Währungsfonds (IWF) sagen inzwischen für 2020 eine globale Rezession mit einem weltweiten Wirtschaftswachstum von -3 % voraus, und für das Bruttoinlandsprodukt (BIP) der Eurozone erwartet der IWF einen Rückgang von 7,5 %. Die WTO prognostiziert, dass der Welthandel 2020 im optimistischen Fall um 12,9 % einbrechen wird. All diese Prognosen sind mit enormen Unsicherheiten behaftet. Je nachdem, wann die Pandemie eingedämmt werden kann, könnte der Rückgang der Wirtschaftstätigkeit und des Welthandels auch weitaus dramatischer ausfallen. In ihrem pessimistischen Szenario sieht die WTO einen Rückgang des Welthandels um 31,9 % voraus, vergleichbar wäre dies nur mit der Großen Depression der 1930er Jahre.

Gleichzeitig haben die Auswirkungen der Pandemie die Fronten zwischen China und den USA weiter verschärft. China übt sich in einer „Maskendiplomatie", indem es publikumswirksam Schutzkleidung exportiert, und schürt Gerüchte, dass das Virus US-amerikanischen Ursprungs sei. Währenddessen deklariert US-Präsident Trump die Krankheit als „chinesisch" und setzt Zahlungen an die Weltgesundheitsorganisation (WHO) aus, da die Organisation zu viel chinesischem Einfluss ausgesetzt sei (Hass und Dong, 2020; Trump, 2020). Es tobt nicht nur ein Kampf um die Deutungshoheit über die Krise; sie hat auch außerhalb der USA protektionistische Reflexe geweckt. Schon als COVID-19 noch als ein vor allem chinesisches Problem wahrgenommen wurde, wurde der Ausfall chinesischer Zulieferer bereits genutzt, um für eine Repatriierung von Wertschöpfungsketten aus China zu werben (García-Herrero, 2020). Nun, da auch in Europa und den USA die ökonomische Aktivität weitgehend zum Erliegen gekommen ist, werden tatsächliche Mängel an medizinischem Material sowie befürchtete Knappheit von Lebensmitteln genutzt, um gegen die Globalisierung zu argumentieren. So verlangte Franksreichs Präsident Macron die „Wiedererlangung der französischen Unabhängigkeit in der Landwirtschaft, dem Gesundheitswesen, der Industrie und der Technologie" (Macron, 2020). Vertreter der Industrie gehen noch weiter und fordern „sanitäre Unabhängigkeit", womit gemeint ist, dass vollständige Wertschöpfungsketten wieder nach Europa verlegt werden sollen (Hudson, 2020). Peter Navarro, der Wirtschaftsberater des US-Präsidenten, fordert die Schaffung ganzer Wertschöpfungsketten in den USA (Politi, 2020). Es wurden in vielen Ländern, inklusive der EU und den USA, Exportkontrollen von medizinischen Gütern eingeführt, die insbesondere kleinere und ärmere Länder vor eine schwierige Situation stellen.

Dies erschwert die Diskussion um die Zukunft des Welthandelssystems und die Suche nach Lösungen gegen den Verfall der internationalen Wirtschaftsordnung. Dabei war der Welthandel schon vor der Corona-Pandemie im Rückzug. Seit der Finanz- und Wirtschaftskrise 2007 ist der Anteil des Handels am globalen BIP zurückgegangen. Dies hatte verschiedene Gründe, technologische und politische, hatte jedoch vor allem aber mit den Auswirkungen der Finanzkrise zu tun (Darvas, 2020; Hoekman, 2015).

## Deutschland und die EU im Handelskonflikt

Die EU befindet sich inmitten des chinesisch-US-amerikanischen Konfliktes, und ist direkt und indirekt von diesem betroffen (Jean et al., 2018). So wurden erste europäische Güter direkt von den USA mit Zöllen belegt und weitere Zölle, insbesondere für den gerade für Deutschland wichtigen Automobilsektor, angedroht. Zölle werden von der US-amerikanischen Regierung inzwischen auch als Drohmittel außerhalb von Handelsstreitigkeiten benutzt (EUObserver, 2020). Indirekt betrifft der Handelskrieg Europa aber auch dadurch, dass die Zölle und Sanktionen, mit denen chinesische Produzenten belegt werden, auch Auswirkung auf ihre europäischen Geschäftspartner haben.[1]

Am größten ist jedoch das Risiko, dass die USA das internationale Handelssystem in Richtung eines Systems von staatlich gelenktem Handel (managed trade) führen. Dies würde bedeuten, dass anstatt von Angebot und Nachfrage Staatsregierungen die gehandelten Mengen bestimmen würden. Anstelle von Markteffizienz würden politische Abwägungen den internationalen Handel leiten. Große Ökonomien wie die USA könnten mit geopolitischen Mitteln andere Länder unter Druck setzten, Güter politisch wichtiger Produzenten zu kaufen. Der chinesisch-US-amerikanische Handelsvertrag gibt einen Vorgeschmack hierfür. Darin wurde festgelegt, dass

1 Für eine Übersicht von EU-China Handel: Dadush et al., 2019.

China 19,5 Billionen US-$ zusätzlich an landwirtschaftlichen Gütern aus den USA einführen muss (Landwirte stellen eine für die Republikaner wichtige Wählerschaft dar). Dies ist zum Nachteil nicht nur Chinas und der meisten US-Bürger, die im Gegenzug höhere Preise für chinesische Güter zahlen müssen, sondern auch anderer Exporteure dieser Güter, insbesondere Brasiliens (Bown, 2020). Weiterhin verhindert es, dass die Vorteile von Freihandel durch komparative Vorteile und Spezialisierung realisiert werden.

Insbesondere Deutschland, als eines der vom Welthandel am stärksten abhängigen Länder der EU, hat ein Interesse daran, dass institutionelle Lösungen für diesen Konflikt gefunden werden. Die Geschichte internationaler Institutionen im Allgemeinen und der WTO im Besonderen zeigt aber, dass diese sich nur extrem langsam fortentwickeln, und dass ohne die Zustimmung der USA nur sehr wenig zu erreichen ist. Viele der Vorstellungen der EU für eine Reform des Handelssystems scheinen auf absehbare Zeit nicht realisierbar. Stattdessen muss eine Agenda entwickelt werden, wie Europa seine Interessen in einem stärker durch Großmachtkonflikte geprägten Handelsumfeld vertreten kann.

## Handelsinstitutionen und die Zukunft der WTO

Die US-Administration scheint beschlossen zu haben, dass die Regeln der WTO ihrem Vorgehen Chinas gegenüber hinderlich sind, und dass sie ihren Konflikt mit China weitgehend außerhalb des institutionellen Rahmens der WTO austragen.[2] Die EU hingegen ist aufgrund ihrer Beschaffenheit weniger agil in ihrer Außenpolitik und hat Multilateralismus und regelbasiertes Handeln in ihrer DNA. Daher ist die Priorität der EU, die WTO zu reformieren und den Konflikt zwischen den USA und China zu entschärfen.

Leider scheint dies ein struktureller Konflikt zu sein, denn auch unter den US-amerikanischen Demokraten wird China als neuer Gegenspieler wahrgenommen. Selbst wenn der Handelskrieg beendet werden sollte, wird eine Wiederherstellung des Status quo ante bellum kaum möglich sein. Langjährige fundamentale Vorbehalte der USA gegen zwischenstaatliche Organisationen haben sich mit dem Vorwurf gepaart, dass internationale Institutionen von China zu seinem Vorteil ausgenutzt werden. So gehen z. B. die US-amerikanischen Vorbehalte gegen den Appellate Body, der letzten Instanz des Schiedsgerichtshofs für zwischenstaatliche Konflikte in der WTO, bis auf seine Gründung zurück.[3] Die ersten Blockierungen der Benennung von Richtern durch die USA, die im Dezember 2019 letztendlich zur Lahmlegung des Appellate Body führten, erfolgten unter Präsident Obama.

Die EU ist als großer „dritter Block" zu bezeichnen, da sie es als einzige Ökonomie von der Größe her mit China und den USA aufnehmen kann. Jedoch fehlen der EU viele der wirtschafts- und außenpolitischen Kompetenzen, um außerhalb von Handelsfragen mit dem gleichen Nachdruck wie China und die USA zu agieren. Gerade deshalb ist die Institutionalisierung des internationalen Handels auch jenseits von seinen grundsätzlichen Vorzügen so wichtig für die EU. Sie erlaubt es, Handelsfragen in einem relativ engen Rahmen innerhalb von EU-Kompetenzen zu erörtern. Wenn jedoch Handelspolitik mit außen- und sicherheitspolitischen Erwägungen verbunden wird, fehlen der EU die formellen Kompetenzen, um ihre Interessen wirksam durchzusetzen.

Viele kleinere Länder, die nicht das wirtschaftliche Gewicht der EU haben, sind noch viel abhängiger als diese von einer regelbasierten internationalen Wirtschaftsordnung, in der nicht das Recht des Stärkeren vorherrscht. Daher versucht die EU in einigen Bereichen eine „Koalition der Willigen" zu bilden und z. B. durch multilaterale Abkommen den von den USA lahmgelegten Appellate Body zu ersetzen.[4] Leider sind viele dieser Länder aber auch direkt wirtschaftlich oder militärisch von den USA abhängig. Insbesondere Chinas aggressive Politik im Südchinesischen Meer hat dazu geführt, dass Länder wie Japan, Südkorea oder Taiwan sich stärker vom militärischen Schutz der USA abhängig sehen (Council on Foreign Relations, 2020). Daher wird die Bereitschaft dieser Länder, eine Koalition mit der EU gegen die Interessen der USA einzugehen, nur sehr begrenzt sein. Außerhalb von einigen, weniger politisierten, Bereichen, wie z. B. E-Commerce, gibt es wenig Hoffnung, dass es zu grundlegenden Reformen der Struktur des Welthandels kommen wird.

Um ihre handelspolitischen Interessen zu erreichen, wird die EU weiterhin stark auf bilaterale Handelsabkommen setzen müssen. In den letzten Jahren hat die EU bereits eine beeindruckende Zahl solcher Abkommen abgeschlossen. Aber gerade gegen die größeren Abkommen wird in Europa häufig opponiert. Themen, die im Grunde nicht direkt mit dem Abbau von Handelsbarrieren zu tun haben, sind immer stärker in den Fokus gerückt:

2 Für einer Übersicht der offiziellen amerikanischen Beschwerden: United States Trade Representative, 2020.

3 Für eine Diskussion des Konfliktes um den Appellate Body: Hufbauer et al., 2018.

4 Für die Vorschläge der EU vgl. Multi-Party Interim Appeal Arbitration Arrangement Pursuant to Article 25 of the DSU (JOB/DSB/1/Add.12).

Umwelt- und Arbeitnehmerschutz (sogenannte „Level Playing Field"-Klauseln) sowie die institutionelle Struktur von Investitionsschiedsverfahren (ISDS). Die Einbindung dieser Themen in Handelsabkommen erlaubt es der EU, ihre politische Agenda in diesen Themen international voranzutreiben und um Rückhalt für diese Abkommen unter den EU-Bürgern zu werben. Dies trägt aber auch das Risiko in sich, dass Handelsabkommen überfrachtet werden. Insbesondere die „Level Playing Field"-Klauseln werden von den Vertragspartnern der EU teilweise als Bevormundung und Protektionismus wahrgenommen.

Viele Vorschläge auf der Agenda der EU, die das institutionelle Gefüge der WTO verbessern könnten, werden mittelfristig nicht umsetzbar sein. Der Weg zu einer weitgehenden Reform des Appellate Body scheint bis auf Weiteres versperrt. Auch die Erfolgschancen für die Bildung eines internationalen Investitionsgerichtshofs für ISDS sehen eher schlecht aus. Viele dieser Vorschläge gehen in die Richtung einer weiteren „Institutionalisierung" des Welthandels, was auf erheblichen Widerstand in den USA stößt. Diese sehen sich von solchen Institutionen in ihrem Handeln beschränkt, haben aber auch ernstzunehmende Einwände hinsichtlich deren Legitimation. Während die Mitgliedstaaten der EU es gewohnt sind, nationale Souveränität mit multilateralen Institutionen zu teilen, sehen die USA dies als undemokratische Einschränkung der Kompetenzen des US-Kongresses, der US-Gerichte und der US-Exekutive.

## Diskussion zur Zukunft des Welthandels

Der Welthandel steht durch den USA-China-Konflikt an einer Wegscheide, und durch die SARS-CoV-2-Pandemie ist die Unsicherheit noch weiter gestiegen. Ob sich der Handelskrieg noch weiter verschärft, wird nicht nur davon abhängen, wer die US-Präsidentschaftswahl 2020 gewinnt, sondern auch davon, wie schnell sich die verschiedenen Regionen von der Pandemie erholen. Sollten sich pessimistische Szenarien für den Westen bewahrheiten, könnte dies zu weiteren sozio-ökonomischen Verwerfungen führen, die die EU und die USA schwächen und zu einer stärkeren Rückkehr zum Protektionismus führen. Auf der anderen Seite kann es sein, dass es in China zu einer schweren Rezession kommt, die das politische System dort infrage stellt. Es wäre zu hoffen, dass die Staaten die Krise als das erkennen, was sie im Grunde ist: ein externer Schock enormen Ausmaßes, der global und koordiniert bekämpft werden sollte. Bisher machen jedoch die Exportbeschränkungen von Medizinprodukten eher den gegenteiligen Eindruck.

Auch wenn die Politik gerade „auf Sicht" agieren muss, so bleiben die langfristigen Herausforderungen der europäischen Handelspolitik bestehen. Um Europas wirtschaftspolitische Interessen wirksam zu vertreten, ist es wichtig Außen- und Sicherheitspolitik stärker zu koordinieren und zu europäisieren. All dies bedeutet nicht, dem Multilateralismus den Rücken zu kehren. Dieser stellt einen hohen Wert an sich dar und sollte auch weiterhin die Grundlage europäischer Außen- und Handelspolitik sein. Jedoch müssen wir anerkennen, dass auf absehbare Zeit eine Reform des Welthandelssystems eher unwahrscheinlich ist. Dieses droht sogar weiter zu erodieren. Deshalb muss die EU strukturelle Schwächen abbauen, die die europäische Verhandlungsposition gegenüber China aber auch den USA angreifbar machen (Leonard et al., 2019; Dadush und Wolff, 2019). Dies betrifft insbesondere die Stärkung des Euro als internationale Währung durch die Vollendung der Kapitalmarktunion, eine stärkere Förderung digitaler Unternehmen durch mehr Forschung und Entwicklung und der Schaffung eines echten digitalen Einheitsmarktes, aber auch die Entwicklung einer europäischen Sicherheitsarchitektur, die von den USA unabhängiger ist. Auch die strategische Kontrolle von Auslandsinvestitionen in Schlüsselindustrien und ein konsequentes Vorgehen gegen staatliche Markteingriffe aus China sollten Teil der Antwort sein.

Was jedoch vermieden werden sollte, ist zu versuchen, durch Protektion von Industrien oder durch Aufweichung des Wettbewerbsrechts „Europäische Champions" zu erschaffen. Dies stärkt die europäische Position im internationalen Handel nicht und würde die multilateralen Institutionen zusätzlich unterwandern. Die unabhängige Wettbewerbskontrolle ist eine Stärke Europas, da sie Monopole unterbindet und effiziente Unternehmen hervorbringt, die auch auf den Weltmärkten Bestand haben (Philippon, 2019). Das Gleiche gilt für die offenen Märkte in Europa, da sie eine Einbindung anderer Länder in die Wirtschaft ermöglichen und Effizienzgewinne durch Spezialisierung und Wettbewerb schaffen. Daher sollte Europa protektionistischen Reflexen widerstehen und auch in diesen schwierigen Zeiten für einen regelbasierten offenen Welthandel eintreten.

Niclas Frederic Poitiers, Ph.D., ist Research Fellow bei Bruegel in Belgien.


Literatur

Bown, C. (2020), Unappreciated hazards of the US-China phase one deal, PIIE Trade and Investment Policy Watch, Januar.
Council on Foreign Relations (2020), Top Conflicts to Watch in 2020: An Armed Confrontation in the South China Sea, 10. Januar.
Crabtree, J., Y. Jie and M. Schneider-Petsinger (2019), US-China Strategic Competition — The Quest for Global Technological Leadership, Chatham House Research Paper.
Dadush, U., M. Domínguez-Jiménez und T. Gao (2019), The State of China-European Union Economic Relations, Bruegel Working Paper, 12.
Dadush, U. und G. Wolff (2019), The European Union’s response to the trade crisis, Bruegel Policy Contribution, 5.
Darvas, Z. (2020), Resisting deglobalisation: the case of Europe, Working Paper, 01/2020, Bruegel.
EUObserver (2020), Trump threatened EU-tariffs over Iran, Germany confirms, 17. Januar.
García-Herrero, A. (2020), Companies must move supply chains further from China, Nikkei Asian Review, 26. Februar.
Hass, R. and K. Dong (2020), The US, China and Asia after the pandemic: more, not less, tension, Brookings, 1. April
Hoekman, B. (Hrsg.) (2015), The Global Trade Slowdown: A New Normal, VoxEU.org eBook.
Hudson, P. (2020), L’Europe doit construire sa souveraineté sanitaire, LesEcho, 28. März.
Hufbauer, G. C., T. Payosova und J. J. Schott (2018), The Dispute Settlement Crisis in the World Trade Organization: Causes and Cures, Peterson
Institute for International Economics, Policy Brief, 18–5, März 2018.
Jean, S., P. Martin und A. Sapir (2018), International trade under attack: what strategy for Europe?, Bruegel Policy Contribution, 12.
Leonard, M., J. Pisani-Ferry, E. Ribakova, J. Shapiro und G. Wolff (2019), Redefining Europe’s economic sovereignty, Bruegel Policy Contribution, 9.
Macron, E. (2020), Rede an die Nation, 13. April.
Philippon, T. (2019), The Great Reversal: How America Gave Up on Free Markets, Harvard University Press.
Politi, J. (2020), US trade adviser seeks to replace Chinese drug supplies, Financial Times, 12. Februar.
Trump, D. (2020), Press Briefing, 14. April.
United States Trade Representative (2019), Report to Congress on China’s WTO Compliance, März 2020.
